# Awareness of Non-alcoholic Fatty Liver Disease in Makkah, Saudi Arabia: A Cross-Sectional Study

**DOI:** 10.7759/cureus.114542

**Published:** 2026-08-14

**Authors:** Khadijah A Ergesous, Rose M Albugami, Miaad A Aljaafari, Layan A Alotaibi, Bayan M Alshuqayfi, Abeer S El Moursy Ali

**Affiliations:** 1 Faculty of Medicine, Umm Al-Qura University, Makkah, SAU; 2 Department of Pathology, Makkah Colleges, Makkah, SAU

**Keywords:** cross-sectional study, makkah population, nafld awareness, non-alcoholic fatty liver disease, public health education

## Abstract

Background: Non-alcoholic fatty liver disease (NAFLD) is a chronic liver condition characterized by excessive fat accumulation in hepatocytes, affecting individuals globally. With a prevalence of 32% among adults, NAFLD is linked to obesity, metabolic disorders, and type 2 diabetes mellitus (T2DM), yet awareness remains disproportionately low, reaching as low as 4.4% in some developed countries like the US, despite its high prevalence. In Saudi Arabia, specifically the Makkah region, data regarding public awareness are scarce. This study aims to bridge this gap by assessing NAFLD awareness and its predictors among Makkah residents.

Methods: A cross-sectional study was conducted using an electronic questionnaire (Google Forms) distributed through social media platforms, with 716 participants (aged ≥18 years) included, who are adult residents of Makkah, Saudi Arabia. The questionnaire gathered demographic information and assessed NAFLD knowledge. The survey instrument was self-constructed; while formal psychometric validation was not conducted, content validity was ensured through review by academic faculty experts. Data were analyzed using RStudio (version 4.3.1). Descriptive statistics, Pearson’s chi-square, and Fisher’s exact tests were utilized. Multivariable logistic regression was performed to identify significant predictors of awareness, with results reported as odds ratios (ORs) and 95% confidence intervals (CIs).

Results: Of the 716 participants, 80% were female, and 54.1% were aged 18-25 years. Only 30.3% were aware of NAFLD, primarily through the Internet (59.9%). Awareness of symptoms was critically low (17.9%). Significant predictors included higher education (p = 0.010) and older age (p = 0.038). Notably, participants with a family history of NAFLD were 32.7 times more likely to be aware of the condition (OR = 32.7, p < 0.001).

Conclusion: The study reveals significant gaps in NAFLD awareness and knowledge among Makkah residents. Given that the internet is the leading source of information, we recommend launching interactive social media campaigns led by trusted healthcare professionals to provide simplified, evidence-based content. In addition, increasing local research and conducting awareness days in public places are essential steps. These practical actions will bridge the knowledge gap, support early detection, and help prevent serious liver complications.

## Introduction

Non-alcoholic fatty liver disease (NAFLD) is a serious chronic liver condition characterized by excessive accumulation of fat (triglycerides) within the cytoplasm of hepatocytes. It is one of the most common liver diseases, affecting individuals across various age groups [1]. NAFLD is strongly associated with cardiometabolic conditions, such as obesity, insulin resistance, type 2 diabetes mellitus (T2DM), hypertension, and dyslipidemia, which are considered major risk factors for its development [2]. NAFLD includes a spectrum of conditions ranging from simple fatty liver to non-alcoholic steatohepatitis (NASH), which can progress to fibrosis and liver damage [3,4]. The estimated global prevalence of NAFLD among adults is 32% and is higher among males (40%) compared to females (26%). The global prevalence of NAFLD has increased over time, from 26% in studies from 2005 or earlier to 38% in studies from 2016 or beyond [5].

According to a study done in 2023, they found that the pooled prevalence of NAFLD in Saudi Arabia was 37.2%, and 58.0% of them had T2DM, which means that T2DM is a major risk factor for NAFLD in Saudi Arabia [6]. Even though the high rate of affected individuals reached 30% globally, the level of awareness about it is still extremely low. A study done in the United States with 11,700 adult individuals revealed that the overall prevalence of liver disease awareness among adults with NAFLD was 4.4%, with the highest prevalence among females (5.3%) and the lowest among males (3.7%) [3]. Given the high and rising prevalence of NAFLD globally and in Saudi Arabia, together with the very low levels of disease awareness reported in previous studies, there is an urgent need to understand how well the public recognizes NAFLD and its consequences.

Assessing awareness in Makkah, a major urban and religious center that receives large numbers of visitors, can help identify knowledge gaps and inform targeted educational and preventive strategies at the community level. Therefore, this study aimed to assess the level of awareness and knowledge of NAFLD among adult residents of Makkah, Saudi Arabia.

## Materials and methods

This cross‑sectional study targeted adult residents of Makkah, Saudi Arabia. A non‑probability convenience sampling technique was used, whereby the electronic questionnaire link was disseminated through social media platforms (e.g., WhatsApp, Twitter, and Instagram), and participants were encouraged to share the link within their networks. Inclusion criteria were age 18 years or older, current residence in Makkah, and ability to read Arabic and provide informed consent. Individuals who were younger than 18 years, not residing in Makkah, or who submitted incomplete questionnaires were excluded from the analysis.

During the data collection period, 740 questionnaire responses were received, of which 24 were excluded due to incomplete responses. The final analytic sample therefore comprised 716 participants. We did not perform a formal a priori sample size calculation using a population‑based formula; instead, the sample size was determined by the number of completed responses obtained within the data collection period. We acknowledge that calculating a minimum required sample size for the Makkah population (for example, assuming a 5% margin of error and appropriate confidence level) would have strengthened the precision and generalizability of the estimates, and this will be taken into account in future research.

The questionnaire included 16 questions that gathered two sets of information: social and demographic data and participants' knowledge about NAFLD (see Appendix).

The study protocol was approved by the Biomedical Research Ethics Committee of Umm Al-Qura University (HAPO-02-K-012-2024-10-2247). Participant confidentiality was maintained throughout the study.

Statistical analysis

All statistical analyses were conducted using RStudio software (R version 4.3.1, Posit PBC, Massachusetts, USA). Descriptive statistics were used to summarize demographic characteristics and awareness levels of participants. Categorical variables were presented as frequencies and percentages.

Analyses were based on available data; participants with missing responses for a given variable were excluded from the corresponding analysis, and no imputation was performed.

Inferential analyses were performed using Pearson’s chi-squared test and Fisher’s exact test to examine associations between awareness of NAFLD and participant characteristics. For multivariable analysis, logistic regression was used to identify significant predictors of NAFLD awareness and awareness of NAFLD symptoms. Odds ratios (ORs) with 95% confidence intervals (CIs) were reported for each variable. A p-value of less than 0.05 was considered statistically significant.

## Results

Demographic characteristics and clinical history

A total of 716 participants were included in the study. The majority of participants were female (80.0%, n = 573), while males accounted for 20.0% (n = 143). The largest age group was between 18 and 25 years (54.1%, n = 387), followed by participants aged 26 to 45 years (30.3%, n = 217). Most participants were Saudi nationals (94.4%, n = 676). Regarding educational levels, the majority held a Bachelor's degree (63.7%, n = 456). In terms of occupation, nearly half of the participants were students (49.6%, n = 355), while 30.4% (n = 218) were employed. The proportion of participants with chronic diseases was 21.6% (n = 155), with diabetes being the most common chronic condition (31.6%, n = 49), followed by asthma (23.9%, n = 37) and hypertension (23.2%, n = 36). Only 4.9% (n = 35) reported having a family history of NAFLD, with a median (IQR) age of NAFLD relatives of 50.0 years (40.0 to 60.0). The most common causes of NAFLD among the family were High levels of triglycerides and bad cholesterol (70.6%, n=24) and obesity (50.0%, n = 17) (Table 1).

**Table 1:** Demographic characteristics of the participants *The variable had five missing records. ¥ The variable had one missing record (n (%)).

Characteristic	Description
Gender	
Male	143 (20.0%)
Female	573 (80.0%)
Age	
18 to 25	387 (54.1%)
26 to 45	217 (30.3%)
> 46	112 (15.6%)
Nationality	
Saudi	676 (94.4%)
Non-Saudi	40 (5.6%)
Educational level	
Primary to secondary	209 (29.2%)
Bachelor's	456 (63.7%)
Master's to Doctorate	51 (7.1%)
Occupation	
Student	355 (49.6%)
Employee	218 (30.4%)
Unemployed	82 (11.5%)
Retired	38 (5.3%)
Other	23 (3.2%)
Chronic disease	155 (21.6%)
If yes, type of chronic disease	
Asthma	37 (23.9%)
Obesity	21 (13.5%)
Diabetes	49 (31.6%)
Hypertension	36 (23.2%)
Others	39 (25.2%)
Family history of NAFLD	35 (4.9%)
If yes, the age of the affected person*	50.0 (40.0 - 60.0)
If yes, causes of NAFLD¥	
Aging	6 (17.6%)
Family history	12 (35.3%)
High levels of triglycerides and bad cholesterol	24 (70.6%)
Hypertension	6 (17.6%)
Metabolic syndrome	5 (14.7%)
Some medications	6 (17.6%)
Type 2 diabetes	12 (35.3%)
Insulin resistance	15 (44.1%)
Obesity	17 (50.0%)

Characteristics of the participants' awareness and knowledge regarding NAFLD

Among the participants, 30.3% (n = 217) reported having heard about NAFLD. Of those who had heard of the disease, the most common source of information was the Internet, reported by 59.9% (n = 130), followed by doctors, seminars, and health campaigns (37.8%, n = 82). Regarding knowledge of NAFLD symptoms, 17.9% (n = 128) of the participants were aware of the symptoms. Among those, fatigue was the most commonly reported symptom (72.7%, 93), followed by jaundice (60.2%, n = 77) and abdominal pain (51.6%, n = 66). In terms of complications, 26.1% (n = 187) were aware that NAFLD could lead to liver cirrhosis, while 19.6% (n = 140) recognized liver failure as a possible complication. Other complications mentioned included liver cancer (11.0%, n = 79) and hepatic failure (19.6%, n = 140) (Table 2).

**Table 2:** Characteristics of participants’ awareness and knowledge regarding non-alcoholic fatty liver disease (NAFLD)

Characteristic	Description
Ever heard about NAFLD	217 (30.3%)
If yes, the main source of information about NAFLD	
Doctors / seminars and health campaigns	82 (37.8%)
Encyclopedia	32 (14.7%)
Television	19 (8.8%)
Internet	130 (59.9%)
I don't have enough information	18 (8.3%)
Others	19 (8.8%)
Have information about disease symptoms	128 (17.9%)
If yes, symptoms	
Abdominal pain	66 (51.6%)
Jaundice	77 (60.2%)
Itching	31 (24.2%)
Swelling	34 (26.6%)
Weight loss	37 (28.9%)
Fatigue	93 (72.7%)
General weakness	50 (39.1%)
Loss of appetite	41 (32.0%)
Complications of NAFLD	
Liver cancer	79 (11.0%)
Liver cirrhosis	187 (26.1%)
Liver failure	140 (19.6%)
Splenomegaly	37 (5.2%)
n (%)	

Factors associated with NAFLD awareness

In the inferential analysis, a significant association was found between educational level and ever having heard about NAFLD (p = 0.010). Among participants with a Master’s or a Doctorate degree, 49.0% (n = 13) reported being aware of NAFLD, compared to 29.4% (n = 134) of those with a Bachelor's degree and 27.8% (n = 58) of those with primary to secondary education. Being ever heard about NAFLD increased with age (p = 0.038), with 38.4% of participants aged >46 years having ever heard (n = 43) compared to 32.7% among those aged 26 to 45 years (n = 71) and 26.6% among those aged 18 to 25 years (n = 103). A family history of NAFLD was also significantly associated with awareness (p < 0.001). An overwhelming 94.3% (n = 33) of participants with a family history of NAFLD were aware of the disease, compared to only 27.0% (n = 184) of those without a family history. In the multivariable regression analysis, family history of NAFLD remained the most significant predictor of awareness. Participants with a family history of NAFLD were over 32.7 times more likely to be aware of the disease compared to those without a family history (OR = 32.7, 95% CI, 10.6 to 162, p < 0.001; Table 3).

**Table 3:** Factors associated with NAFLD awareness Analyses were conducted on available cases; participants with missing data for any variable in the model were excluded from the corresponding analysis (listwise deletion), which accounts for minor differences in denominators compared with Table 1. No data imputation was performed. *NAFLD: non-alcoholic fatty liver disease; OR: odds ratio; CI: confidence interval

Characteristic	Ever heard about NAFLD			Multivariable regression		
	No (N = 499)	Yes (N = 217)	p-value	OR	95% CI	p-value
Gender						
Male	95 (66.4%)	48 (33.6%)	0.343			
Female	404 (70.5%)	169 (29.5%)				
Age						
18 to 25	284 (73.4%)	103 (26.6%)		Reference	Reference	
26 to 45	146 (67.3%)	71 (32.7%)	0.038	1.19	0.81, 1.75	0.362
> 46	69 (61.6%)	43 (38.4%)		1.05	0.62, 1.74	0.853
Nationality						
Saudi	473 (70.0%)	203 (30.0%)	0.506			
Non-Saudi	26 (65.0%)	14 (35.0%)				
Educational level						
Primary to secondary	151 (72.2%)	58 (27.8%)		Reference	Reference	
Bachelor's	322 (70.6%)	134 (29.4%)	0.010	0.99	0.68, 1.44	0.940
Master's to Doctorate	26 (51.0%)	25 (49.0%)		1.81	0.89, 3.64	0.100
Occupation						
Student	263 (74.1%)	92 (25.9%)				
Employee	141 (64.7%)	77 (35.3%)				
Unemployed	56 (68.3%)	26 (31.7%)	0.072			
Retired	22 (57.9%)	16 (42.1%)				
Other	17 (73.9%)	6 (26.1%)				
Chronic disease						
No	393 (70.1%)	168 (29.9%)	0.689			
Yes	106 (68.4%)	49 (31.6%)				
Family history of NAFLD						
No	497 (73.0%)	184 (27.0%)	<0.001	Reference	Reference	<0.001*
Yes	2 (5.7%)	33 (94.3%)		32.7	10.6, 162	

Factors associated with awareness of NAFLD symptoms

In the inferential analysis, a significant association was found between educational level and awareness of NAFLD symptoms (p = 0.013). Among participants with a Master’s or a Doctorate degree, 31.4%% (n = 16) were aware of the symptoms, compared to 18.2% (n = 83) of those with a Bachelor's degree and only 13.9% (n = 29) of those with a primary education. A family history of NAFLD was also significantly associated with awareness of symptoms (p < 0.001). Among participants with a family history of NAFLD, 71.4% (n = 25) were aware of NAFLD symptoms, compared to only 15.1% (n = 103) of those without a family history. In the multivariable regression analysis, family history of NAFLD was the most significant predictor of awareness of NAFLD symptoms. Participants with a family history of NAFLD were over 12 times more likely to be aware of the symptoms compared to those without a family history (OR = 12.4, 95% CI, 5.87 to 27.0, p < 0.001) (Table 4).

**Table 4:** Factors associated with having information about disease symptoms NAFLD: non-alcoholic fatty liver disease

Characteristic	Awareness of NAFLD symptoms			Multivariable regression		
	No (N = 588)	Yes (N = 128)	p-value	OR	95% CI	p-value
Gender						
Male	118 (82.5%)	25 (17.5%)	0.891			
Female	470 (82.0%)	103 (18.0%)				
Age						
18 to 25	319 (82.4%)	68 (17.6%)				
26 to 45	182 (83.9%)	35 (16.1%)	0.371			
>46	87 (77.7%)	25 (22.3%)				
Nationality			0.718			
Saudi	556 (82.2%)	120 (17.8%)				
Non-Saudi	32 (80.0%)	8 (20.0%)				
Educational level						
Primary to secondary	180 (86.1%)	29 (13.9%)	0.013	Reference	Reference	
Bachelor's	373 (81.8%)	83 (18.2%)		1.26	0.80, 2.05	0.324
Master's to Doctorate	35 (68.6%)	16 (31.4%)		2.03	0.92, 4.31	0.077
Occupation						
Student	293 (82.5%)	62 (17.5%)				
Employee	181 (83.0%)	37 (17.0%)				
Unemployed	68 (82.9%)	14 (17.1%)	0.112			
Retired	25 (65.8%)	13 (34.2%)				
Other	21 (91.3%)	2 (8.7%)				
Chronic disease						
No	468 (83.4%)	93 (16.6%)	0.084			
Yes	120 (77.4%)	35 (22.6%)				
Family history of NAFLD						
No	578 (84.9%)	103 (15.1%)	<0.001	Reference	Reference	<0.001*
Yes	10 (28.6%)	25 (71.4%)		12.2	5.89, 27.0	

## Discussion

NAFLD is a significant health issue that is becoming more common globally. Saudi Arabia is among the countries with a high prevalence of NAFLD, yet there is a lack of research on public awareness regarding this condition [7].

The current study investigated the awareness of NAFLD among residents of Makkah, Saudi Arabia. The data presented in the study indicate that 30.3% of participants in the current study reported having prior knowledge of the condition. In comparison, a similar study conducted in Jazan, Saudi Arabia, in 2024, found that 53% had general awareness. This significant disparity underscores the diversity of awareness levels among populations and the importance of doing targeted research to systematically eliminate knowledge gaps [8]. Researchers evaluated Mexican-American women through community-based surveys and interviews, discovering similar findings [9]. Meanwhile, a Chinese study found that 5% of participants were aware of NAFLD. Furthermore, another study conducted in Saudi society discovered that only 7% of participants had a solid comprehension [7]. These results highlight the necessity of customized educational initiatives to systematically address knowledge gaps and enhance public health outcomes [9].

It was clear that the Internet was the most popular source of knowledge regarding NAFLD among participants, accounting for 59.9%. Similarly, a Mexican study found that many participants identified the Internet as their primary source of information regarding NAFLD, especially those with a family history of liver disease [9]. In addition to online sources, healthcare experts were important, with 37.8% of participants citing them as a main source of knowledge. However, research in Western nations (Brooklyn, New York, USA) found that the majority of participants said their doctors had never mentioned NAFLD to them [10]. In addition, a study conducted among youth in the United States revealed that instructional strategies like films and written materials were effective in increasing awareness of the value of family medical history, enabling people to more accurately assess their health risks. Furthermore, in the same study, 4% of those who learned about NAFLD stated that the Internet was their primary source of information. This illustrates how important online learning environments are becoming to people's education [11].

Consistent with previous evidence, our findings add to growing data that NAFLD is common in Saudi Arabia and in the wider Middle East and North Africa region. A recent systematic review estimated a pooled NAFLD prevalence of 16.8% among adults in Saudi Arabia, increasing to 58.0% among those with type 2 diabetes, while global analyses suggest that the MENA region has among the highest regional prevalence estimates worldwide (around 35-37%) compared with a global average near 30% [6].

The findings of this study reveal important insights into the awareness and knowledge of NAFLD among the population of Makkah, Saudi Arabia. One of the key findings is the lack of a statistically significant relationship between gender and awareness of NAFLD. Despite the majority of participants being female (80.0%, n = 573), gender did not influence awareness levels. This is consistent with findings from other studies, such as a US-based study where gender was not a significant predictor of NAFLD awareness [3]. Similarly, age did not show a significant association with awareness, with the largest age group being 18 to 25 years (54.1%, n = 387). This aligns with previous research indicating that demographic factors like age and gender may not be primary determinants of health awareness in certain populations [7,8].

Nationality, however, emerged as a significant factor influencing NAFLD awareness. Non-Saudi participants (5.6% of the sample) demonstrated a higher awareness rate (35%) compared to Saudi nationals (30%). This discrepancy suggests that non-Saudi individuals may have better access to health information, possibly due to differences in educational or cultural exposure. A prior study in Saudi Arabia found that university-educated Saudis had significantly higher awareness of NAFLD (92%) compared to non-Saudis with similar educational backgrounds (8%) [8]. These findings highlight the need for tailored awareness initiatives in Makkah to address disparities in health knowledge and ensure equitable access to information for all residents.

Educational qualifications also played a role in shaping awareness, although the association was not statistically significant. Participants with advanced degrees (Master’s or Doctorate) were more likely to be aware of NAFLD, which aligns with studies emphasizing the role of education in health literacy [9,10]. However, the most significant predictor of awareness was having a family history of NAFLD (OR = 32.7, p < 0.001). This finding is consistent with research showing that personal or familial exposure to a disease significantly enhances awareness and understanding [11]. For example, a study in Mexico found that individuals with a family history of liver disease were more likely to seek information about NAFLD [12]. This underscores the importance of leveraging personal and familial experiences in public health campaigns to improve disease awareness.

Regarding knowledge of NAFLD causes, participants were most aware of obesity (50.0%), high triglyceride and LDL cholesterol levels (70.6%), and insulin resistance (44.1%). However, awareness of other risk factors, such as certain medications, was limited. This is consistent with findings from a study in Pakistan, where only 32% of participants were knowledgeable about the main causes of chronic liver disease [13]. Other previous studies suggested that there was a positive attitude toward understanding the role of obesity and high levels of blood cholesterol as causes of NAFLD [14,15]. The lack of broader awareness highlights the need for comprehensive educational programs that address all potential risk factors for NAFLD.

Awareness of NAFLD symptoms was notably low, with only 17% of participants recognizing symptoms such as fatigue, abdominal discomfort, and jaundice. This is likely due to the asymptomatic nature of early-stage NAFLD, which has been reported in other studies as a barrier to early detection [16,17,18]. Similarly, awareness of complications like liver cirrhosis (26.1%) and liver failure (19.6%) was limited, emphasizing the urgent need for targeted educational initiatives to improve public understanding of NAFLD and its potential consequences.

Limitations

This cross‑sectional study cannot establish causal relationships between participant characteristics and NAFLD awareness. Data were obtained using a self‑constructed, self‑reported electronic questionnaire, which, despite being developed from the literature and reviewed by experts, was not formally validated and may be subject to measurement and response bias.

Recruitment through social media using convenience sampling likely under‑represented older adults and individuals with limited internet access; the predominance of young (54.1% aged 18-25 years) and female participants (80%) further limits generalizability to the wider Makkah population. No formal a priori sample size calculation was undertaken; instead, the final sample reflected all completed questionnaires, which may have reduced the precision of some estimates.

In addition, a small amount of missing data led to listwise exclusion in the regression analyses, potentially reducing statistical power, and detailed socioeconomic or cultural determinants of NAFLD awareness were not assessed. Nevertheless, the study identifies important gaps in NAFLD awareness and supports the need for targeted, culturally appropriate educational interventions in the region.

## Conclusions

This study highlights significant gaps in NAFLD awareness and knowledge among Makkah residents. While factors, such as nationality and family history, play a crucial role in shaping awareness, broader educational efforts are needed to address the low levels of recognition of NAFLD symptoms and complications. Given that the internet is the leading source of information, we recommend launching interactive social media campaigns led by trusted healthcare professionals to provide simplified, evidence-based content. In addition, increasing local research and organizing NAFLD-focused awareness days in public places are essential steps. These practical actions may help bridge the knowledge gap, support early detection, and prevent serious liver complications in Makkah and beyond.

## Appendices

Study questionnaire

استبيان عن قياس معرفة سكان مكة المكرمة عن مرض الكبد الدهني غير الكحولي (NAFLD)

ندعوك للمشاركة في دراسة بحثية تتعلق بقياس مستوى المعرفة والوعي بمرض الكبد الدهني غير الكحولي (NAFLD)  في مكة المكرمة. يتم إجراء هذه الدراسة بواسطة مجموعة من الطالبات من كلية الطب بجامعة أم القرى، بإشراف ] د/ عبير شاكر[ المشاركة في هذه الدراسة تطوعية، وستساعد مشاركتك على فهم أفضل لمستوى الوعي والمعرفة حول الكبد الدهني غير الكحولي في المجتمع. سيتم الحفاظ على المعلومات التي تشاركنا إياها بسرية تامة. إذا كان لديك أي أسئلة حول هذه الدراسة، يرجى التواصل مع مشرفة البحث [د/ عبير شاكر،[(abeershaker72@gmail.com) بإكمال هذا الاستبيان، فإنك تقر بموافقتك على المشاركة في هذه الدراسة.

We invite you to participate in a research study on awareness of non-alcoholic fatty liver disease (NAFLD) in Makkah, Saudi Arabia.

This study is being conducted by a group of students from Umm Al-Qura College of Medicine, under the supervision of Dr. Abeer Shaker.

Participation in this study is voluntary, and your participation will help us better understand the level of awareness and knowledge about non-alcoholic fatty liver disease in the community.

The information you share with us will be kept strictly confidential.

If you have any questions about this study, please get in touch with the research supervisor (Dr. Abeer Shaker, (abeershaker72@gmail.com)). By completing this questionnaire, you agree to participate in this study.

هل توافق على المشاركة في البحث؟ *

نعم

لا

Do you agree to participate in the research? *

Yes

No

الجنس *

أنثى

ذكر

Gender *

Female

Male

العمر *

١٨-٢٥

٢٦-٣٦

٣٧-٤٥

فوق ٤٦

Age *

18-25

26-36

37-45

Over 46

الجنسية *

سعودي

غير سعودي

Nationality

Saudi

Non-Saudi

المستوى التعليمي *

ابتدائي

متوسط

ثانوي

بكالوريوس

ماجستير

دكتوراة

Education level

Primary school

Intermediate/middle school

High School

Bachelor's degree

Master's degree

Doctorate (PhD)

المهنة*

طالب

موظف

غير موظف

متقاعد/ة

أخرى

Occupation *

Student

Employed

Unemployed

Retired

Other

هل تعاني من أحد الأمراض المزمنه؟*

نعم

لا

Do you suffer from any chronic diseases? *

Yes

No

اذا كانت الأجابة نعم الرجاء تحديد النوع (يمكنك اختيار أكثر من إجابة)

السكري

الضغط

الربو

السمنة

لايوجد

أخرى

If the answer is yes, please specify the type (you can choose more than one answer)

Diabetes

Hypertension (high blood pressure)

Asthma

Obesity

None

Other

هل سمعت عن مرض الكبد الدهني غير الكحولي (NAFLD)؟*

نعم

لا

Have you ever heard of non-alcoholic fatty liver disease (NAFLD)? *

Yes

No

إذا كانت الإجابة بنعم, ماهو  مصدر معلوماتك الرئيسي عن الكبد الدهني غير الكحولي (NAFLD)؟ (يمكنك اختيار أكثر من إجابة)

الإنترنت

الأطباء / الندوات والحملات الصحية

التلفاز

دائرة المعارف

ليس لدي معلومات كافية

أخرى

If the answer is yes, what is your main source of information about Non-Alcoholic Fatty Liver Disease (NAFLD)? (You can choose more than one answer)

Internet

Doctors / Seminars and Health Campaigns

Television

Encyclopedia / General Knowledge

I do not have enough information

Other

هل يعاني أي من أفراد عائلتك من مرض الكبد الدهني الغير كحولي (NAFLD) ؟*

نعم

لا

Does any member of your family suffer from Non-Alcoholic Fatty Liver Disease (NAFLD)? *

Yes

No

إذا وجد شخص مصاب كم يبلغ من العمر ؟

إجابتك

  If there is an affected person, how old are they?

Your answer:

اذا اجبت بنعم، فما هي  الأسباب الاخرى التي قد تؤدي إلى المرض؟(يمكنك اختيار أكثر من إجابة)

السمنه

مقاومة الأنسولين

ارتفاع نسبة الدهون الثلاثية والكوليسترول الضار

مرض السكري من النوع الثاني

ارتفاع ضغط الدم

متلازمة التمثيل الغذائي

بعض الأدوية

التقدم في العمر

التاريخ العائلي

If you answered yes, what are the other causes that may lead to the disease? (You can choose more than one answer)

Obesity

Insulin resistance

High triglycerides and bad cholesterol

Type 2 diabetes

High blood pressure

Metabolic syndrome

Certain medications

Advancing age

Family history

هل لديك معلومات عن أعراض المرض؟*

نعم

لا

Do you have information about the symptoms of the disease? *

Yes

No

إذا كانت الإجابة بنعم ماهي الأعراض (يمكنك اختيار أكثر من إجابة)

التعب و الإرهاق

فقدان الشهية

الضعف العام

آلام في البطن

الاصفرار

التورم

الحكة

فقدان الوزن

If the answer is yes, what are the symptoms? (You can choose more than one answer)

Fatigue and exhaustion

Loss of appetite

General weakness

Abdominal pain

Yellowing (Jaundice)

Swelling

Itching

Weight loss

هل تعلم ماهي المضاعفات المستقبلية التي يمكن أن تصيب الشخص نتيجة اصابته بمرض الكبد الدهني غير الكحولي                     (يمكنك اختيار أكثر من إجابة)

تليف الكبد

الفشل الكبدي

تضخم الطحال

السرطان الكبدي

لا اعلم

Do you know what future complications can affect a person as a result of having non-alcoholic fatty liver disease? (You can choose more than one answer)

Liver fibrosis/cirrhosis

Liver failure

Splenomegaly (Enlarged spleen)

I don't know
